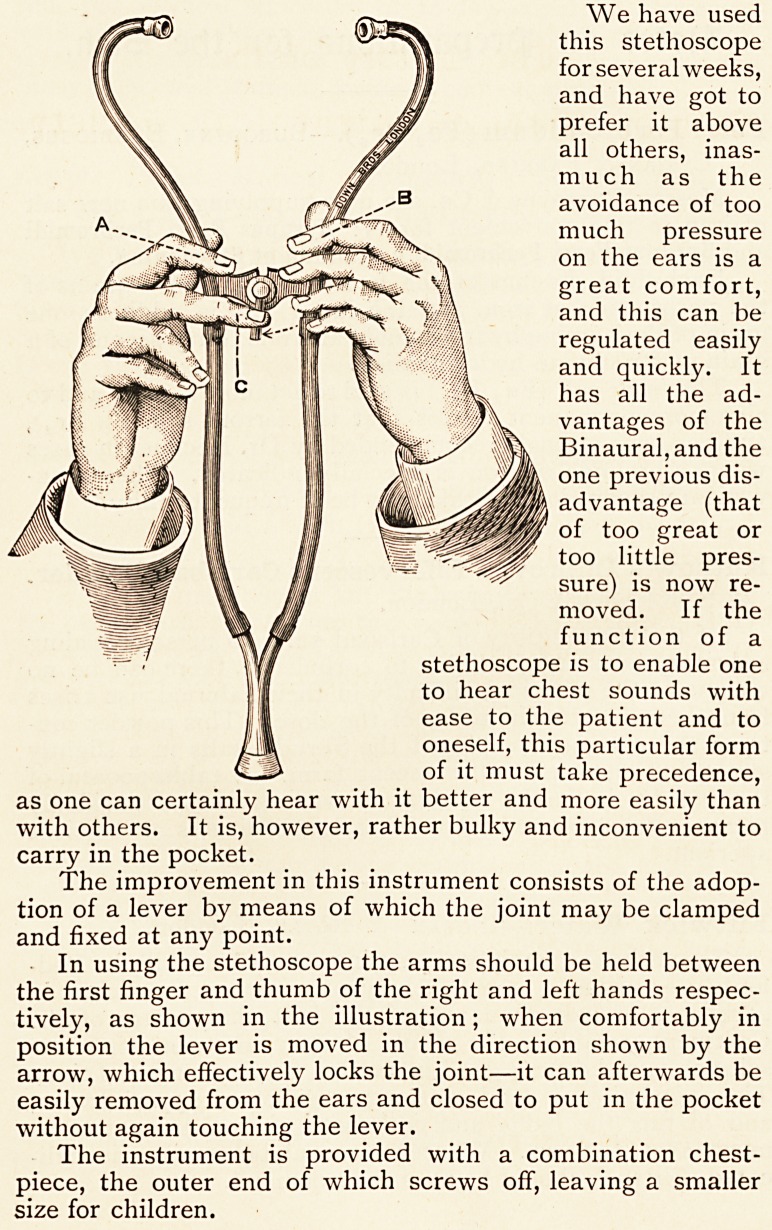# Notes on Preparations for the Sick

**Published:** 1891-09

**Authors:** 


					Botes on preparations for tbe
Ferri Perbromidum (Fe2 Br0).?Burgoyne, Burbidges,
Cyriax & Farries, London.
Messrs. Burgoyne & Co. are now supplying this new salt
of iron in two convenient forms: Syrupus Ferri Perbromidi
and Syrupus Ferri Perbromidi c. Quinina et Strychnina.
Each fluid drachm of the former contains five grains of
the perbromide of iron, and the latter has in addition one
grain of the quinine hydrobromide and one thirty-second of a
grain of strychnine hydrobromide.
The ferric salt (Fe? Brc) is said to act more rapidly and to
give more permanent results that the ferrous salt (Fe Br2),
and has been strongly recommended by Dr. Hecquet in cases
where a bromide of iron is specially indicated. These con-
venient preparations are likely to be of frequent utility.
Kutnow's Improved Effervescent Carlsbad Powder.
S. Kutnow & Co., London.
Of the great utility of Carlsbad salts to persons leading
sedentary lives and inclining to corpulence, there can be no
question ; the principal difficulty in their habitual use arises
from the nauseous character of the dose. This powder con-
tains the active principles of the Sprudel salts in a slightly
sweetened and feebly effervescent form. A tablespoonful of
the powder in half a tumbler of water makes a decidedly
pleasant draught, and taken in early morning is an efficient
aperient.
Flitwick Water.?Ingram & Royle, London.
The Flitwick chalybeate spring, near Ampthill, in Bed-
fordshire, yields a unique - chalybeate water, far richer in
carbonate of iron than any of the best chalybeates of the
Continent, retaining the whole of its constituents in clear
solution, and keeping good in bottles for any length of time.
The proportion of solid matter per gallon is 255.26 grains,
and of this the oxide and carbonate of iron amount to as
much as 144 grains. Wherever a chalybeate water is indi-
cated, Flitwick should be at once in demand.
214 PREPARATIONS FOR THE SICK.
Dr. Herschell's Improved Binaural Stethoscope.?
Down Bros., London.
We have used
this stethoscope
for several weeks,
and have got to
prefer it above
all others, inas-
much as the
avoidance of too
much pressure
on the ears is a
great comfort,
and this can be
regulated easily
and quickly. It
has all the ad-
vantages of the
Binaural, and the
one previous dis-
advantage (that
of too great or
too little pres-
sure) is now re-
moved. If the
function of a
stethoscope is to enable one
to hear chest sounds with
ease to the patient and to
oneself, this particular form
of it must take precedence,
as one can certainly hear with it better and more easily than
with others. It is, however, rather bulky and inconvenient to
carry in the pocket.
The improvement in this instrument consists of the adop-
tion of a lever by means of which the joint may be clamped
and fixed at any point.
In using the stethoscope the arms should be held between
the first finger and thumb of the right and left hands respec-
tively, as shown in the illustration; when comfortably in
position the lever is moved in the direction shown by the
arrow, which effectively locks the joint?it can afterwards be
easily removed from the ears and closed to put in the pocket
without again touching the lever.
The instrument is provided with a combination chest-
piece, the outer end of which screws off, leaving a smaller
size for children.
We have used
stethoscope
II for several weeks,
U and have got to
M prefer it above
Af all others, inas-
/w much as the
a?/b avoidance of too
<2^ much pressure
on the ears is a
\\ great comfort,
' ") "\ and this can be
lrV ^'v^'4 regulated easily
III W and quickly. It
C 111 ftv ^as ^ie ac^~
1? X. 'wk. vantages of the
11 Binaural, and the
Mh\ yjlkk one previous dis-
II jim^h advantage (that
If of too great or
If ^00 little pres-
^JggllBff M if ' ^ sure) is now re-
^?L M moved. If the
^kfir function of a
^ Iff stethoscope is to enable one
1,1 to hear chest sounds with
1 ease to the patient and to
JJI oneself, this particular form
of it must take precedence,
as one can certainly hear with it better and more easily than
with others. It is, however, rather bulky and inconvenient to
carry in the pocket.
The improvement in this instrument consists of the adop-
tion of a lever by means of which the joint may be clamped
and fixed at any point.
In using the stethoscope the arms should be held between
the first finger and thumb of the right and left hands respec-
tively, as shown in the illustration; when comfortably in
position the lever is moved in the direction shown by the
arrow, which effectively locks the joint?it can afterwards be
easily removed from the ears and closed to put in the pocket
without again touching the lever.
The instrument is provided with a combination chest-
piece, the outer end of which screws off, leaving a smaller
size for children.

				

## Figures and Tables

**Figure f1:**